# Constancy and diversity in the flavivirus fusion peptide

**DOI:** 10.1186/1743-422X-5-27

**Published:** 2008-02-14

**Authors:** Stephen J Seligman

**Affiliations:** 1 Department of Microbiology and Immunology, New York Medical College, Valhalla, New York, USA

## Abstract

**Background:**

Flaviviruses include the mosquito-borne dengue, Japanese encephalitis, yellow fever and West Nile and the tick-borne encephalitis viruses. They are responsible for considerable world-wide morbidity and mortality. Viral entry is mediated by a conserved fusion peptide containing 16 amino acids located in domain II of the envelope protein E. Highly orchestrated conformational changes initiated by exposure to acidic pH accompany the fusion process and are important factors limiting amino acid changes in the fusion peptide that still permit fusion with host cell membranes in both arthropod and vertebrate hosts. The cell-fusing related agents, growing only in mosquitoes or insect cell lines, possess a different homologous peptide.

**Results:**

Analysis of 46 named flaviviruses deposited in the Entrez Nucleotides database extended the constancy in the canonical fusion peptide sequences of mosquito-borne, tick-borne and viruses with no known vector to include more recently-sequenced viruses. The mosquito-borne signature amino acid, G104, was also found in flaviviruses with no known vector and with the cell-fusion related viruses. Despite the constancy in the canonical sequences in pathogenic flaviviruses, mutations were surprisingly frequent with a 27% prevalence of nonsynonymous mutations in yellow fever virus fusion peptide sequences, and 0 to 7.4% prevalence in the others. Six of seven yellow fever patients whose virus had fusion peptide mutations died. In the cell-fusing related agents, not enough sequences have been deposited to estimate reliably the prevalence of fusion peptide mutations. However, the canonical sequences homologous to the fusion peptide and the pattern of disulfide linkages in protein E differed significantly from the other flaviviruses.

**Conclusion:**

The constancy of the canonical fusion peptide sequences in the arthropod-borne flaviviruses contrasts with the high prevalence of mutations in most individual viruses. The discrepancy may be the result of a survival advantage accompanying sequence diversity (quasispecies) involving the fusion peptide. Limited clinical data with yellow fever virus suggest that the presence of fusion peptide mutants is not associated with a decreased case fatality rate. The cell-fusing related agents may have substantial differences from other flaviviruses in their mechanism of viral entry into the host cell.

## Background

Flaviviruses are in the family *Flaviviridae *and are usually arthropod-borne. They are enveloped, single-stranded, positive sense RNA viruses that contain about 11,000 nucleotides. Translation results in a polyprotein that is co- and post-translationally modified to produce three structural (C, M and E) and seven non-structural proteins (NS1, NS2a, NS2b, NS3, NS4a, NS4b and NS5). More than 75 flaviviruses have been named. The most common virulent flaviviruses are the mosquito-borne dengue (four serotypes, DENV1–4), Japanese encephalitis (JEV), yellow fever (YFV), West Nile (WNV) and St. Louis encephalitis (SLEV) and the tick-borne encephalitis viruses. A proposed classification assigns the tick-borne encephalitis viruses to three groups: mammalian tick-borne virus (TBEV), seabird tick-borne virus and Kadam [1].

Particular concerns with flaviviruses are the increasing number of dengue infections in which infection with a second serotype greatly increases the chance of developing dengue hemorrhagic fever and dengue shock syndrome, the continuing western spread of JEV from the Far East across Asia, the persistence of YFV especially in Africa and the introduction of WNV into the Western Hemisphere in 1999. In addition to vector-borne species, the genus *Flavivirus *includes viruses with vertebrate hosts but no known vector (NKV).

The currently unclassified flavivirus cell-fusing related viruses (CFRV) have been isolated only from mosquitoes or insect cell lines and contain cell fusing agent virus (CFAV) [2,3], Kamiti River virus (KRV) [4] and Culex flavivirus (CXFV) [5]. The first CFRV virus described, a CFAV, had the property that on propagation in *Aedes albopictus *cells, the predominant cytopathic effect was cell fusion, a finding not necessarily found with other cell lines or with other CFRV. The observation that in measles virus, loss of cell fusing (giant cell formation) ability can be the result of a single mutational event suggests that a similar relatively minor change in the flaviviral genome could change the cell-fusing phenotype [6].

Protein E, glycosylated in most flaviviruses, enables receptor-mediated attachment of the virion to the host cell and fusion with host cell membrane. It contains the principal epitopes eliciting neutralizing antibodies. The surface of the virion has 90 copies of the protein E dimer [7]. Solution of the crystal structure of the soluble portion of E indicates that the protein can be divided into 3 domains, I, II and III [8]. The crystallographic study reveals a "cd" loop at the tip of the elongation domain, domain II. The loop was interpreted to be the fusion peptide. In the mature virion, part of the fusion peptide of one monomer in the dimer pair is buried under the surface of domains I and III in the adjacent monomer.

Previously, on the basis of conservation of amino acids among flavivirus species, a high glycine content, molecular flexibility and chemical characteristics similar to known fusion peptides, it had been suspected that amino acids 98–120 of protein E mediated fusion [9]. Subsequent estimates of the fusion peptide included amino acids 98–110 [10] and 99–116 [11]. The crystallographic study demonstrates that the cd loop contained amino acids 98–113 [8]. Functional assessments of only some of the amino acids influencing fusion or growth have been reported e.g. for amino acids 104 [12,13], 106 [13], and 107 [12-14]. In part because it is the start of a conserved amino acid sequence and because of its participation in a salt bridge with K110, the N-terminus of the fusion peptide is likely to be D98. The C-terminus has not been defined functionally. Conservation of the dipeptide 112 SI 113 in all of the pathogenic flaviviruses except the four dengue serotypes and the inclusion of those amino acids in the cd loop suggest that they be included as the C-terminus. In the present study the fusion peptide was considered to be the 16 amino acids in the cd loop.

A canonical sequence is defined in the current report as a sequence conserved in a variety of viruses as contrasted with a wild-type sequence characterizing an individual virus. According to this definition, 12 of the 16 amino acids in the flavivirus fusion peptide comprise a canonical sequence, 98 DRGWGNXCGXFGKGXX 113 (with X representing variable amino acids). In mosquito-borne flaviviruses, amino acid 104 is G and in tick-borne strains, 104 is H. With either vector, 107 is L, except in the tick-borne Powassan (POWV) in which it is F.

Since it is located in the envelope protein in tight association with the adjacent monomer, the flavivirus fusion peptide has been classified as mediating class II fusion with host cell membranes [15]. As a consequence of viral entry into a cell via clathrin-dependent endocytosis, the virion becomes encased in an endosome. During maturation the endosome becomes acidic causing release of the buried tip of the fusion peptide. The protein E monomer now associates as a trimer and fusion with host cell membrane occurs, permitting transfer of the RNA genome into the host cell cytoplasm. The requirement for extensive conformational change in the fusion peptide coupled with its high degree of conservation among the flaviviruses suggests that fusion peptide mutants would be rare.

The current report was initiated to investigate whether the conservation of flavivirus fusion peptide was preserved in the more recent isolates especially in strains not yet associated with disease, to evaluate diversities associated with mutations in the wild-type sequences and to study the homologous sequence in the CFRV.

## Results

### Inter-virus constancy: The canonical fusion peptide sequence

The wild-type sequences of the pathogenic arthropod-borne flaviviruses are depicted (Table 1). By virtue of the definitions used in this study, they all had the canonical sequence. Some other flaviviruses also had the canonical sequence (Table 2) with the expected G104 or H104 depending on their vector. Only with Kadam, a tick-borne virus for which a separate grouping has been proposed [1], was there an amino acid (N) other than G or H at codon 104. Both the NKV and the CFRV had the mosquito-borne signature amino acid. Indeed the entire canonical virus sequence with the mosquito-borne signature was found in some NKV (Rio Bravo, Apoi [16] and Montana myotis leukoencephalitis virus [17]). However, other NKV, Modoc [18] and the more distantly related Tamana bat virus [19], although maintaining the mosquito-borne signature, had altered fusion peptides.

**Table 1:** Wild-type fusion peptide sequences in pathogenic arthropod-borne flaviviruses

Flavivirus	Wild-type sequence^a^
Mosquito-borne except dengue^b^	DRGWGNGCGLFGKGSI
Dengue type 1	DRGWGNGCGLFGKGS**L**
Dengue type 2	DRGWGNGCGLFGKG**GI**
Dengue type 3	DRGWGNGCGLFGKG**SL**
Dengue type 4	DRGWGNGCGLFGKG**GV**
Tick-borne^c ^except Powassan	DRGW**H**NGCGLFGKGSI
Powassan including deer tick	DRGW**H**NGCG**F**FGKGSI

^a^Changes from the amino acids in the most common mosquito-borne sequence are in bold

^b^JEV, YFV, WNV including Kunjin, Murray Valley encephalitis and SLEV viruses

^c^TBEV, Langat, louping ill, Omsk hemorrhagic fever virus, Kyasanur Forest disease virus including Alkhurma, Royal Farm virus, Karshi, Gadgets Gully, Spanish sheep encephalomyelitis and Turkish sheep encephalitis viruses.

**Table 2:** Wild-type fusion peptide sequences in other flaviviruses

Flavivirus	Vector	Wild-type sequence^a^
Additional mosquito-borne not in Table 1^b^	Mosquito	DRGWGNGCGLFGKGSI
Viruses with variations only in amino acid 16^c^	Mosquito	DRGWGNGCGLFGKGS**X**
Iguape virus	Mosquito	DRGW**N**NGCGLFGKGS**L**
Bussuquara	Mosquito	**N**RGW**N**NGCGLFGKG**D**I
Seabird tick-borne viruses^d^	Tick	DRGWGN**H**CGLFGKGSI
Kadam virus	Tick	DRGWGN**N**CGLFGKGSI
Some NKV^e^	None known	DRGWGNGCGLFGKGSI
Modoc virus	None known	DRGWGNGC**A**LFGKGSI
Tamana bat virus^f^	None known	DRGW**DS**GC**FI**FGKG**EV**
Cell fusing agent (CFAV)	No known vertebrate host	**N**RGWG**T**GC**FKW**G**I**G**FV**
CFA Puerto Rico (CFAV)	No known vertebrate host	**N**RGWG**T**GC**FKW**G**I**G**FV**
Culex flavivirus (CXFV)	No known vertebrate host	**N**RGWG**T**GC**FKW**G**I**G**FV**
Kamiti River virus (KRV)	No known vertebrate host	**N**RGWG**T**GC**FEW**G**L**G**QV**

^a^Changes from the amino acids in the most common mosquito-borne sequence are in bold.

^b^Including Usutu, Sepik and Entebbe bat, Ilheus, Bagaza and Yokose viruses

^c^Kokobera, Rocio, Kédougou and Zika viruses in which amino acid 16, X, = M, L, Y and L respectively

^d^Tyuleniy, Meaban and Saumarez Reef viruses

^e^Rio Bravo, Apoi and Montana myotis leukoencephalitis viruses

^f^A new genus has been proposed for this virus.

### Inter-virus variation: The cell-fusion related viruses

**Figure 1:**
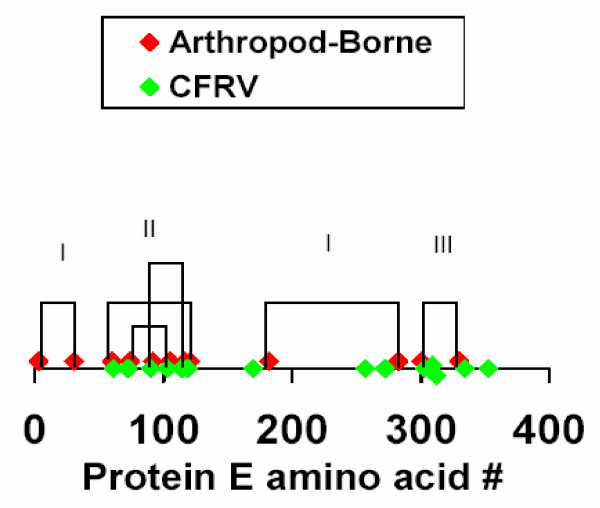
**Distribution of conserved cysteines in arthropod-borne and CFRV flavivirus protein E**. The disulfide linkages and their domains are indicated for the arthropod-borne flaviviruses. The figure demonstrates that 4 of the CFRV conserved cysteine residues (257, 309, 312 and 352) are not homologous to the arthropod-borne flaviviruses.

CFAV [2,3], KRV [4] and CXFV [5], together constituting the CFRV, have been grown only in mosquitoes or insect cell lines. As was noted in the original description of CFAV, the sequence homologous to the fusion peptide contains only 8 amino acids in common with the canonical flavivirus fusion peptide [2]. The present analysis extended the identity of the homologous peptide to CXFV but found that the KRV differed from the other CFRV in 3 fusion-peptide related amino acids (Table 2). Five of the six conserved glycine residues were maintained in all CFRV. The amino acid changes in the CFRV abolished both ends of the salt bridge between amino acids 98 and 110. Another difference previously noted is that the CFAV has 14 cysteines in protein E rather than 12 as in flaviviruses [2]. The current findings demonstrated that the 14 cysteines were conserved in all CFRV. One pair of cysteines in domain I, four pairs in domain II (including the fusion-peptide related pair) and one pair in domain III were present in both the arthropod-borne flaviviruses and the CFRV (Figure 1). However, the arthropod-borne flaviviruses had two disulfide-linked cysteines at the beginning of domain I. The CFRV lacked these conserved cysteines, but had four additional conserved cysteines, presumably linked by disulfide bonds, whose pairing has not been determined. Accordingly, there are likely to be distinct differences in the folding pattern of their protein Es, possibly resulting in a changed cell-surface orientation.

### Variation within viruses: Amino acid mutations in flavivirus fusion peptides

The average prevalence of mutations in pathogenic flaviviruses was 5.0% (Table 3). In YFV, 27% of the fusion-peptide sequences contained mutations. In the other arthropod-borne flaviviruses capable of causing disease in humans, the percentage varied from 0 to 7.4. The relative lack of mutations of YFV in amino acids 108–113 could be related to the fact that 80% of the YFV sequences came from one laboratory whose reverse primers do not permit detection of mutants in the sequence FGKGSI, the six C-terminal acids of the fusion peptide [20-24]. On the other extreme, although only 97 sequences have been deposited, no fusion peptide mutants have been found with SLEV (p = 0.11 comparing SLEV with pathogens other than YFV), a flavivirus associated in general with comparatively few amino acid changes either in nature or on laboratory passage [25,26]. No deviations from wild-type sequences were found in flaviviruses other than those listed in Table 1. Although only relatively few sequences have been deposited, the lack of deviation from the wild-type sequences also included the CFRV in which the current analysis found 1 sequence for the original CFAV, 1 for CFAV (Puerto Rico) and 8 for CXFV. All encoded the same amino acid sequence. The 2 sequences deposited for KRV were identical to each other. However, in many of the other viruses listed in Table 2, only one sequence has been deposited. Since the prevalence of mutations in the pathogenic flaviviruses averaged 5% (range 0 to 27%), an appreciable percentage of sequences determined only once may not be wild-type.

**Table 3:** Frequency of nonsynonymous mutations in flavivirus fusion peptides

Flavivirus	Wild-type fusion peptide sequence	# of mutant sequences	Total # of sequences	% with mutations
Dengue 1	DRGWGNGCGLFGKGSL	12	428	2.8
Dengue 2	DRGWGNGCGLFGKGGI	14	413	3.4
Dengue 3	DRGWGNGCGLFGKGSL	2	240	0.8
Dengue 4	DRGWGNGCGLFGKGGV	9	171	5.3
West Nile including Kunjin	DRGWGNGCGLFGKGSI	6	303	2.0
Japanese encephalitis	DRGWGNGCGLFGKGSI	18	243	7.4
Yellow fever	DRGWGNGCGLFGKGSI	47	177	26.6
St. Louis encephalitis	DRGWGNGCGLFGKGSI	0	97	0.0
Tick-borne encephalitis	DRGWGNHCGLFGKGSI	4	151	2.6
Powassan including deer tick	DRGWGNHCGFFGKGSI	1	22	4.6
				
Total		113	2,245	5.0

### Fusion-peptide mutants in yellow fever virus

**Figure 2:**
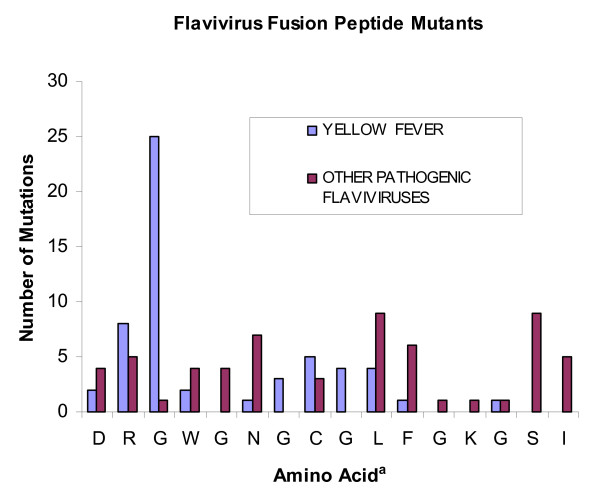
**Comparison of fusion peptide mutations in yellow fever virus with other pathogenic flaviviruses**. ^a^The mosquito-borne sequence is shown. In tick-borne viruses G is replaced by H as the 7^th ^amino acid. In Powassan, a tick-borne virus, the 10^th ^amino acid is F in lieu of L. Amino acids 15 and 16 are SL, GI, SL and GV in DENV1–4, respectively instead of SI.

Comparison of the fusion peptide mutations in other flaviviruses with those reported with YFV revealed that in YFV the predominant mutation was G100S (Figure 2). In YFV 25/56 (45%) of the mutations were at the 100^th ^codon in E (all but one were G100S). In contrast only 1/60 (1.7%) mutations in other pathogenic flaviviruses occurred at codon 100 (p < 0.0001) (Figure 2). Investigators in the laboratory that had deposited 80% of the YFV sequences including 46/47 of the mutant sequences were concerned about the large number of YFV fusion-peptide mutants [24]. They pointed out that at least some of the mutations would result in profound alterations of the structural integrity of the fusion peptide, namely abrogation either of the disulfide bond with mutation of C105 or of the salt bridge between amino acids D98 and K110. They determined the mouse LD_50 _in two mutants, the most commonly encountered, G100S and one that abolished a disulfide bond by the mutation C105S. In both instances there was a 10^6.5 ^reduction in mouse virulence compared to the wild-type sequence. The decreased virulence for mice in the two isolates tested contrasts with the present analysis of their data indicating that six of seven (86%) of Brazilian patients whose virus had fusion peptide mutations died, including all three with the G100S mutation. Since the usual case fatality rate with YFV infection is 20–50%, infection with a fusion-peptide mutated strain did not seem to decrease the risk of a fatal outcome.

**Figure 3:**
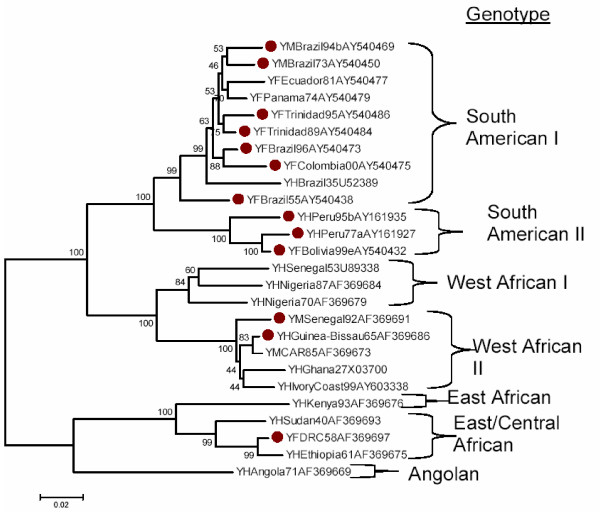
**Phylogenetic tree of yellow fever viruses with a G100S E protein mutation**. The nearest neighbor-joining tree was developed from sequences in the prM/E region (nucleotides 643–1308). Similar results were obtained using maximum parsimony. Selected strains with G100S mutations indicated by red circles were supplemented with strains with the wild-type codon to indicate additional branching patterns.

Because of the frequency of the G100S mutation in YFV, a phylogenetic tree was developed, an abridged version of which is shown (Figure 3). The tree demonstrates that the mutation is widely distributed amongst most of the genotypes (predominantly South American but also three African isolates in two branches), a pattern consistent with a selective advantage conferred by a G100S mutation and either multiple independent mutations or extensive recombination. While recombination appears to occur in dengue, JEV and SLEV, it has not yet been observed with YFV [27]. Thus multiple independent G100S mutations, "convergent evolution", are the most likely explanation for the dispersal of the G100S mutation throughout the tree. Such dispersal is distinctly different from the pattern for a nonsense mutation in DENV1 protein E in which strains with the mutation at codon E248 were all part of the same lineage, a finding most consistent with a unitary origin of the mutation [28].

## Discussion

The current study confirms the well-known conservation in the canonical sequence of the fusion peptide amino acids in pathogenic flaviviruses. In addition, it documents the extent of the conservation and variation in more distantly related flaviviruses. Protein E contains 493–501 amino acids in the arthropod-borne flaviviruses and flaviviruses with no known vector. Only 18% of the amino acids are completely conserved in pathogenic flaviviruses [29]. Although the fusion peptide contains only 3.2% of the amino acids in protein E, it contains 13% of the conserved amino acids in E (p < 0.00001), making it the most conserved sequence in protein E and possibly in the entire flavivirus genome. Twelve of the 16 fusion peptide amino acids are completely conserved not only in pathogenic strains, but also in many flaviviruses found in diverse environments such as bats, rats and seabirds and in flaviviruses with no known vectors. Six of the conserved fusion peptide amino acids are glycines (five in the tick-borne viruses), mostly alternating with another amino acid. Since glycine residues may permit rotation around their C-C and C-N bonds, their presence is likely to facilitate the conformational changes necessary for fusion, including release of the tip of the fusion peptide from underneath domains I and III at the start of fusion. The conservation of fusion-peptide amino acids, in association with the structural studies indicating highly-organized conformational changes accompanying fusion, suggests that there are considerable constraints on evolutionary changes in the fusion peptide, particularly when the virion must be capable of fusing both with mammalian and arthropod cells.

The sequences homologous to the fusion peptide in the CFRV differ in 8 amino acids from the other flaviviruses. In KRV the wild-type sequence (based on 2 determinations) was NRGWGTGCF**E**WG**L**G**Q**V (the three amino acids different from the other CFRV are in bold). The canonical sequence in the other CFRV (based on 10 sequences) was NRGWGTGCF**K**WG**I**G**F**V. These differences from the fusion peptide sequences in the vast majority of flaviviruses, in combination with the differences in the presumed disulfide binding patterns (as shown in Results) suggest that the mechanism of CFRV virus entry into the cell differs from the other flaviviruses.

### Disparity between inter-flaviviral conservation and intra-flaviviral variation of the fusion peptide

Given the considerable conservation in fusion peptide within the otherwise variable E protein, the high prevalence of fusion-peptide mutants in a given flavivirus was surprising. The findings with two flaviviruses are particularly relevant: YFV and DENV1.

In YFV, the high prevalence, 27%, of fusion peptide mutations is noteworthy (Table 3). Limited outcomes data in humans, cited above, suggest that despite laboratory evidence of diminished virulence in two fusion-peptide mutants, patients infected with the mutant strains have a high mortality rate. Dual infection with both a fusion-peptide mutant and wild-type virus is a plausible explanation. Two possible mechanisms for production of two different viral genomes by the same cell should be considered. The first is co-infection with two different viruses [28]. The second is the possibility that the virion contains two different viral genomes, as is regularly the case with retroviruses. But why does YFV stand out as the flavivirus with by far the largest prevalence of fusion peptide mutations? Most of the sequencing on YFV virus material is done on specimens lyophilized for periods of time up to several decades. Consequently the virus is propagated prior to sequencing. The laboratory that deposited 46/47 of the fusion peptide mutants passaged virus at a multiplicity of infection (moi) of 1 (many of the other laboratories did not state the moi in their methods sections). Since this is a relatively high moi and passage with high moi's may encourage the emergence of defective virus, it is possible that the fusion peptide mutants increased in frequency on serial passage. However, it should be noted that the laboratory tried to minimize the number of passages prior to sequencing. On the other hand, it is also possible that if the other laboratories passaged the virus under conditions that selected against persistence of the mutants (such as passage at a low moi), the sequences determined in the other laboratories may have underestimated the prevalence of mutations.

In DENV1, 2.8% of the fusion peptide sequences contained amino acid changes (Table 3). Eleven of the 12 mutant sequences came from a laboratory that did direct sequencing without prior cultivation [28]. Their evaluation of sequence heterogeneity revealed a nonsense codon in the middle of E at codon 248 in 68/290 (23%) of their sequences. They documented expression of a truncated protein E. In some viral populations 11 of the 20 sequences cloned from the same specimen contained the nonsense codon. Since the resulting truncated protein E lacks the C-terminal amino acids necessary for attachment to the viral envelope, none of the protein E and obviously none of the fusion peptide can be attached to the envelope. The authors postulate that co-infection with fully infective virus complements the defective virus. Persistence of the mutation for at least three years and its presence in specimens obtained more than a thousand miles apart was documented. The prolonged persistence of the mutation suggests that it confers a survival advantage to the virus. Moreover, in addition to the mutation resulting in a protein truncated at amino acid 247, analysis of the sequences deposited in GenBank in connection with the DENV1 article revealed at least two sequences with the following mutations: In the fusion peptide itself (N103D), a nonsense mutation at codon 2 and a 72 nucleotide deletion that resulted in loss of the entire fusion peptide (SJS, unpublished). These repetitive mutations occurred in the same specimens ("virus population") supporting the interpretation that they were the result of virus propagation and were not simply dead end mutations or the result of sequencing errors. Accordingly, at least with DENV1, sequence heterogeneity with rescue of defective virus by complementation with fully infective virus is a plausible explanation for the persistence of fusion peptide mutations.

### Quasispecies and sequence heterogeneity

Several reports in addition to the above discussed DENV1 have quantified sequence heterogeneity in flaviviruses including DENV2 [30], DENV3 [31-33] and WNV [34] and a non-quantitative evaluation of nucleotide variations in YFV vaccines [35]. These findings support the possibility that transmission of virus occurs as a combination of sequences and might, at least in part, explain the prevalence of the fusion peptide mutations. Sequence heterogeneity is found with many other RNA viruses and is considered to result from both the lack of a proof-reading mechanism in virus-encoded RNA polymerases and extensive viral multiplication. The variations are usually attributed to virus being transmitted as a quasispecies [36]. The concept of quasispecies as formulated by Eigen is that the virus is propagated not as a single genome, but as a mixture of genomes with the result that there is no single wild-type sequence. In evolutionary terms, survival of the fittest becomes survival of the fittest quasispecies. The bacteriophage Qβ fits the definition of a quasispecies [37]. When cloned isolates are propagated, they revert to a mixture. Analogous results have been obtained in laboratory experiments with TBEV in which mammalian-adapted virus and tick-adapted virus can be purified from isolated plaques that, on additional propagation in the alternate cell type, revert to the other tropism [38].

Despite suggestions that the term quasispecies should be restricted to situations in which specific criteria have been demonstrated, it has become common practice to label the mere presence of sequence variation in a given specimen as a quasispecies. Indeed the sole finding of different virus sequences in the same geographic area has been considered sufficient [39]. However, questions have been raised about the real world applicability of the quasispecies concept in finite populations [40,41]. Proponents of the quasispecies concept believe that the questions have been satisfactorily addressed [42]. But, as even the concept's proponents acknowledge, in instances in which the population has not been sufficiently studied, it is preferable to call the virus population a swarm [43]. By whatever name one calls the finding of sequence diversity, the presence of a variety of genomes transmitted during the course of flavivirus propagation appears to be intimately involved in the explanation for the high prevalence of fusion peptide mutations in most of the pathogenic flaviviruses.

### Questions raised by the current analysis

Is the seventh amino acid in the flavivirus fusion peptide, G in the mosquito-borne viruses and H in the tick-borne strains, both necessary and sufficient in determining the arthropod vector? Evidence against the possibility is the observation that mutant TBEV/DENV4 plasmid constructs containing the genes for TBEV proteins M and E in a DENV4 backbone did not yield viable virus with H104G (i.e. replacing the signature tick-borne amino acid with the mosquito one) [12], However, the double mutant H104G, L107F grew. It may be relevant that the normally tick-borne POWV, whose wild-type sequence contains F107, has been isolated from mosquitoes [44]. It would be interesting to determine the fusion peptide sequences in mosquito isolates from normally tick-borne viruses (and vice versa).

What are the implications of the fusion peptide differences between CFRV and the other flaviviruses? The changes in eight of the 16 amino acids in CFRV in the sequence homologous to the flavivirus fusion peptide bring up the possibilities either that fusion differs substantially in these viruses or that there is another mechanism for the RNA genome to gain entry into the cell. Even if it is assigned to a genus other than flavivirus, the Tamana bat virus fusion peptide sequence with six amino acid changes raises similar questions. In contrast with the CFRV that have no known vertebrate host, the Tamana bat virus has no known vector. Although more isolates need to be sequenced to establish the wild-type, Bussuquara virus and Iguape virus with three and two amino acid changes respectively may be additional candidates for altered cell entry mechanisms. If there is a mechanism for entry of the genome into the cell that does not require fusion, then it might also be utilized by the fusion peptide mutants.

Are there additional insights to be gained by analyzing conserved amino acids in other parts of the viral genome? One example is a conserved glycine in the region between protein E domains I and III, G301 [29]. Could that glycine be necessary for some of the conformational changes in protein E that accompany fusion? What about flavivirus complex-specific determinants? Can amino acids conserved in one flavivirus complex that differ from homologous amino acids conserved in another be helpful guides in elucidating the peptides crucial for complex delineation?

## Conclusion

The contrast between the extensive conservation of the flavivirus fusion peptide sequence between flaviviruses and the high percentage of mutations prevalent in many of the individual flaviviruses is notable. The first result suggests that there are significant constraints on the permitted evolutionary changes in the fusion peptide consistent with the maintenance of pathogenicity in flaviviruses and with the ability to infect both arthropod and mammalian hosts. The high prevalence of fusion peptide mutants is best explained by the presence of dual infection either through superinfection or by virions containing two RNA copies. The limited clinical information on YFV outcomes (a high fatality rate in a few yellow fever patients infected with fusion peptide mutants) is consistent with the hypothesis that, despite demonstration that cloned mutants have diminished mouse virulence, dual infection can result in particularly virulent disease. In fact the persistence of mutants with defective replicative ability (best demonstrated by a nonsense codon in DENV1) suggests that dual infection can confer a replicative advantage. The marked change in the composition of the "fusion peptide" in the CFRV suggests that entry of the viral genome into host cell cytoplasm differs from the other flaviviruses.

## Methods

Mosquito-borne flavivirus nucleotide sequences that contained all of the nucleotides encoding the fusion peptide amino acids were obtained from the NCBI website using the CoreNucleotide option [45]. That site uses the Entrez Nucleotides database, a collection of sequences from several sources, including GenBank. YFV sequences were imported into Mega version 4 [46]. Neighbor-joining phylogenetic trees with 1000 bootstrap iterations were then generated with the Mega program. Similar trees were obtained using maximum parsimony.

The search for fusion-peptide-containing flavivirus sequences for specific arthropod-borne flaviviruses known to be pathogenic was initiated by Blastp searches using the wild-type sequences shown in Table 1 in conjunction with the virus name. Additional tick-borne flaviviruses were identified from a recent publication [1]. In one instance a sequence inadvertently omitted from GenBank was supplied by the author. Mutant sequences were identified starting with sequences with less than perfect matches and ending when the peptides were part of proteins other than flavivirus protein E. Published sources of the sequences were obtained from the sequence citations or, if the citation listed the source as "unpublished", from a search in the NCBI website with the PubMed option.

Additional flaviviruses were identified from a protein search in the NCBI site using the search terms "flavivirus no known vector". However, most of the viruses identified by that search term were mosquito-borne [47]. Completeness of the search for fusion peptide sequences was substantiated by reviewing the listing in the article by Cook et al of all available flaviviral sequences which might include the fusion peptide (genome and polyprotein [[48], their Table 2]. For CFRV the search terms were "cell fusing agent", "Kamiti River virus" and "culex flavivirus".

Categorical variables were evaluated by a two sided Fisher's exact test for p(O>=E|O<=E) [49].

## Competing interests

The author declares that he has no competing interests.
